# Acquiring religious words: dialogical and individual construction of a word's meaning

**DOI:** 10.1098/rstb.2021.0359

**Published:** 2023-02-13

**Authors:** Franziska E. Viertel, Oliver Reis, Katharina J. Rohlfing

**Affiliations:** ^1^ Department of German Studies and Comparative Literacy Studies, Psycholinguistics, Paderborn University, Warburger Straße 100, 33098 Paderborn, Germany; ^2^ Department of Catholic Theology, Paderborn University, Warburger Straße 100, 33098 Paderborn, Germany

**Keywords:** abstract words, religious words, emotional valence, perspectivation, contextualization

## Abstract

By the age of eight, there is a significant increase in abstract words in the child's lexicon. A crucial contribution can be seen in the linguistic input, i.e. the way how abstract words are presented by caregivers by means of linguistic perspectivation and emotionalization. Following an interactionist way, we were interested in how the semantics of abstract words is constructed by child and caregiver in duet. We focused on a subset of abstract words and studied the acquisition of meaning of the religious concept *mercy.* We expected religious words to be emotionally anchored and presented with perspectivation, both contributing to learning. Exploring the dialogic constructions, we investigated eight 7- to 8-year olds and their parents during dialogic reading and studied their strategies focusing on the linguistic means of emotionalization and perspectivation in contextualizing the word. In a subsequent test, we analysed these means used by the children and assessed their individual understanding of *mercy*. Our analyses indicate that during reading, the enrichment of semantics by emotionalization was related between child and caregiver, whereas cross-situationally, a simultaneous enrichment of emotionalization and perspectivation was present. Moreover, the children demonstrated a conceptual understanding of *mercy* in religious contexts, but not in secular contexts.

This article is part of the theme issue ‘Concepts in interaction: social engagement and inner experiences’.

## 1. Introduction: abstract terms as a field of research in religious education and psycholinguistics

In existing studies on abstract words, the underlying concepts that are studied are assumed not only to be known to everybody but also to be ‘abundant in daily conversation’ [1, p. 719]: they refer to ‘diverse concepts’ and are associated with ‘situation-embedded instances'. Instead of having intrinsic properties as concrete concepts, abstract concepts are considered to be rather relational [2] that enables them to be applied widely across situations. However, a subgroup of abstract words that has not been considered so far, are religious words such as *mercy, blessing* or *holy*. Similar to other abstract words, they are situation-embedded as well but require a specific contextualization by a social group that knows their meaning [3]. The subgroup of religious abstract words is attractive because it highlights the fact that a practice of applying the words needs to be established by a social group [4]. So abstract words are not only interesting for psycholinguistic studies on how they are represented and processed but also for other disciplines like religious education.

In research on religious education, there is a long-lasting discussion about the role that religious words have on religious behaviour. With respect to a social group that establishes a practice of how to use religious language, it has been argued on the one hand, that religious language is not bound to specific words, but that religious language emerges in a specific quality of reference to the world [5–7]. On the other hand, studies show that if children and adolescents use religious language at all, their language is integrated in traditional formulae that are religiously influenced in terms of social conventions of faith communities [4,8–10]. At the heart of the debate lies the question of whether religious language opens a transcendent access to the world precisely through these terms or whether a transcendent perspective is necessary to understand and use them. No matter which position is taken, it seems obvious that religious words do not refer to direct entities but their meanings emerge in the social observation of the world. Therefore, we understand these core religious terms, necessary to form religious language and thus a religious approach to the world at all [11], as abstract terms in the sense of (psycho)linguistics [12]. Religious education faces the problem of limited acquisition of religious concepts that can be reconstructed as a question of acquisition of abstract concepts [13–15].

### (a) Mechanisms for enriching the semantics of abstract (religious) words

In the literature, the development of abstract words is reported to be relatively independent of the learner's experience on a sensory or motor level [16]; instead, it mainly unfolds on a linguistic level. Between the ages of 8 and 9 years old, children achieve the largest learning progress in acquiring the meaning of abstract words [17]. According to the situational systematicity approach, abstract words are predominantly anchored in experiences and are acquired in diverse contexts, so that the construction of meaning relies on elements that occur across multiple situations and must be related to each other by the learner [18,19]. In contrast with concrete words, the challenge in learning abstract words is to systematically connect the elements that constitute a concept, which are dislocated across space and time. However, it seems unlikely that children of early primary school age will already be able to recognize all nuances of a concept (also in distinction to similar ones) and extract complex meaning from it, as they still lack the diversity of linguistic contexts, which is often only provided by literacy [20]. For this reason, especially for younger children, other mechanisms of acquisition must exist, in which the initial construction of meaning of abstract words does not emerge so substantially from linguistic information. This means that mechanisms must be at work that enrich the linguistic information so that children in the word-learning process gain an access to the basic concept at all. For example, semantically important constituents of a concept can be perceived more saliently or mental images can be activated through enrichment. In the following, we summarize three keys to the acquisition of abstract words that contribute to their contextualization.

#### (i) Emotionalization

A possible key to the acquisition of abstract concepts in children with insufficient linguistic resources could be found in emotional experiences and associations [16], as they help to anchor the novel meaning more strongly with internal and somatic states (cf. also [21,22]) and to bind them to linguistic representations. This is also evident at brain level: brain areas associated with emotions are more involved in the processing of abstract concepts than in the processing of concrete words [23]. In acquisition, abstract words that refer exclusively to emotional states are learned earlier than those that are not associated with emotions [24,25]. Abstract words that are emotionally valenced––either in a positive or negative way––are acquired earlier than words with a neutral valence [26]. This effect of emotional valence [23] is particularly evident in 8- to 9-year olds and pronounced for abstract words with positive emotional connotations [17,27]. In line with these study findings, Kousta *et al*. [16] and Vigliocco *et al*. [20] suggest a facilitative role of emotion in extracting the meaning of abstract words, insofar as 'emotion may provide building blocks especially for early acquisition of abstract vocabulary' [20, p. 546].

Although it is known that the type of linguistic input is important for building abstract concepts, the exact role of linguistic input from the caregiver remains unclear as well as how it should be structured [28]. In this regard, studies with young children that highlight the positive influence of emotionally enriched maternal speech during shared book reading on children's mental state vocabulary seem promising [29,30].

#### (ii) Perspectivation

Abstract concepts differ from concrete concepts insofar as the view is consciously directed inwards in the sense of a mentalizing process rather than towards visible referents (e.g. *truth* versus *ball*) where the focus is guided towards prototypical features by the senses. The central question that arises at this point is how children manage to focus on the relevant constituents of an abstract concept in order to construct stable and robust concepts. On the one hand, this can only be achieved with competent interaction partners who successfully activate vivid mental images, and who enable the learners to make the constituents of a concept more visible in a figurative sense. For young learners, the path is not solely via definitions of an abstract concept, since these are too abstract, too difficult to imagine and often linguistically too demanding when not embedded in teaching strategies [28]. Instead, it is via the illustration of (real-life) situations, so that an experience is evoked. In this respect, perspectivation is a linguistic means that we consider central. A linguistic perspectivation is the ‘…representation of something for someone from a given position … and it is thus verbalized for (an) addressee for a specific purpose’ [31, p. 37]. In this process, what is verbalized becomes relevant for the learner, which enables him to perceive semantic constituents, and even more: to experience them—an aspect that makes perspectivation a mechanism so fruitful in supporting the acquisition of abstract concepts. For example, a perspectivation is created by providing a direct reference to a situation from the child's experience. On the other hand, perspectivation is primarily about an activation of another's perspective and about enabling the learner to reflect on different perspectives side by side or simultaneously, which we consider important for a widened conceptual knowledge. For example, when reading a story aloud, the caregiver could take on the perspective of a character and convey an emotion from that character's perspective, i.e. she or he focuses on a specific mental or emotional state.

Important prerequisites for being able to follow the caregiver in perspectivation are social-cognitive and linguistic in nature. First, children must be equipped with fundamental social-cognitive competences such as having a Theory of Mind about their fellows that is, they can comprehend what another believes or is convinced of even if this thinking does not correspond to reality or their own beliefs [32]. Second, children need to understand which mental or emotional states a conversational partner is referring to by retrieving the related semantic concepts from the mental lexicon [33]. In the case of emotion words, even 2-year olds already use such terms as *happy*, *sad*, and *mad* in their productive vocabulary [25]. In later development during preschool and early primary school age, children are equipped with a rich knowledge of multiple mental states of others and consequently, may take their perspective [34–36]. In a longitudinal study on 3- to 4-year-olds' knowledge of mental states, the use of mental state words by mothers was positively associated with children's vocabulary growth in this domain [37]. In particular, when mothers used elaborations in the context of a picture story, this often resulted in making the protagonist's perspective more intelligible—in the sense of a perspectivation—and thus prompting the children to perceive another perspective beyond their own. Moreover, a perspectivation may stimulate children to think about non-visible and abstract entities that underlie a behaviour and possibly to reflect on a new concept.

This example shows that emotionalization alone is not sufficient to activate the inner ideas that contribute to a rich conceptual construction of an abstract concept. Additionally, a linguistic perspectivation is necessary, which can be achieved by mental state words [38–40] but also by prosodic means, or subjective comments [41–43] that express cognitive processes, attitudes, preferences, intentions or even emotions (of others).

#### (iii) Interactionist contextualization

While the previous sections focused largely on the caregivers' linguistic input strategies, another line of research draws on the construction of meaning in interactionist duet with the caregiver resulting in an active contribution of the child. Accordingly, a concept is co-constructed in social interactions in an interpersonal way (cf. e.g. [3,44,45]). Especially, for the acquisition of abstract concepts, a mental construction supported by social partners who know the semantics is particularly significant—in contrast with concrete concepts, whose meaning is supported by referents in the situation. For the mental construction of an abstract concept to unfold, it seems beneficial when perspectivation and emotionalization are jointly constructed in interactions instead of being predetermined—as with input strategies [13]. By doing so, the contextualization of a concept is managed mutually. In our view, this will lead to a deeper semantic anchoring as when the caregiver is contextualizing alone. For example, in social interactions, an exchange of different perspectives is taking place, and the caregiver can connect to and develop the child's perspective and link to potentially different or incomplete conceptions of meaning. Conversations about a concept also offer the child the opportunity to ask questions, to express his or her state of understanding, and to demand further elaborations or explanations from the conversational partner, which leads to a further development and differentiation of conceptual knowledge. Such an exchange was found in dialogic reading that is an established routine in many Western cultures [46,47]. Various linguistic studies have shown the significant relation of dialogic reading and language acquisition as well as conceptual development for everyday and abstract concepts (cf. [48–50]). Yet, to this date, to our knowledge, no studies exist that focus on the concrete role of contextualization strategies in the acquisition of abstract concepts in dialogic reading.

### (b) Context-dependent models for the meaning of the abstract religious word *mercy*

To study the role of linguistic input as a contribution to both children's construction of the abstract concept in a dialogue as well as its semantic structure, we chose the religious word ‘mercy [Barmherzigkeit]’. Although an exact translation is quite difficult, the German equivalent of *merciful* is found in *barmherzig*, which in German is composed of two linguistic roots: ‘erbarmen’ and ‘armherzig’. ‘Erbarmen [commiseration]’ has its origin in a situation of need, which not only has to be perceived by the other person, but also requires her to put herself in the shoes of a person in need in the sense of a perspectivation. At the same time, an emotional impulse is triggered that makes one react to the other's distress. ‘Armherzig [poor-hearted]’, in turn, is composed of poor and heart, so that a distressed situation touches another person's heart, which again reflects an act of perspectivation and emotionalization. The concept of *mercy*, thus, is a prototypical religious concept for seeing reality under the eyes of the merciful God and acting on behalf of a certain reality. While in its history *mercy* could be used religiously and secularly, today, it is a word known almost exclusively in religious texts and liturgy. Therefore, other semantic neighbours are used today, even if the impulse to act mercifully is understood intuitively. Another reason to choose *mercy* was that its meaning can be captured by models derived from theological considerations (cf. [51,52]) presuming that mercy of people always presupposes the mercy of God, who forgives sinners and rejoins them in community (table 1, model A), and who turns to the victims and lamenting people (table 1, model B). In both models, people then also act mercifully.

**Table 1.** Models for the meaning of 'mercy'.

model A of forgiveness	model B of loving care
(like merciful God) a merciful person forgives the guilty persons that are now in troubles	(like merciful God) a merciful person is touched by the troubles of another person
**action structure including semantic aspects**	
(1) person A is guilty to a person / God	(1) person A has troubles
(2) person B / God has a legal claim against person A with corresponding sanctions or compensation	(2) person B is touched by the troubles of person A
(3) person A shows remorse and willingness to accept the sanctions or compensation	(3) person B decided to help person A spontaneously
(4) person B / God renounces the sanctions or compensations	(4) person A is free from troubles or the troubles are less
(5) person B / God transforms the situation into a healing one for both	(5) person B expects nothing in return from person A
**context dependent semantic neighbours *kind* and *just***	
** ***just*: close and appropriate	*kind*: close and appropriate
*kind*: close but inappropriate	*just*: close but inappropriate

The underlying idea of theological models is to open up *mercy* in its full complexity [52] and thus, to consider on the one hand, the religious-historical development and, on the other hand, different contexts of origin and use, which in turn activate various nuances of meaning. For this purpose, it is necessary to look at the different contexts (that evoke *mercy*) in isolation and to pinpoint the underlying deep semantic structure stepwise (in the following: *semantic aspects*). At this level, we emphasize the strong interdisciplinary meshing between religious education and psycholinguistics.

Biblically seen, mercy is first regarded as God's nature towards the world, who empathizes with the world, guides it in a healing way and forgives people who have become entangled in guilt. Mercy then becomes the mission of those people who refer to God and are to become similar to him in terms of their actions. In ecclesiastical language, this line of meaning can be traced back to the tenth century and is related to God's expectations and behaviour that is clerically preferred [51,53–55]. Historically seen, Christianity already structurally protects mercy through institutions, e.g. for the care of the poor and the sick [54]. In Church law, too, mercy towards people in need is a core principle itself [56]. However, these models are hardly available today because on the one hand, institutionalized care is associated with caritas and on the other hand, there are hardly any practical references to Church law.

Today, models that focus on the context of interpersonal behaviour are most available. According to Reis [52], for the meaning of mercy, two models can be identified.

In model A, the aspect of guilt or sin is the central starting point, which is forgiven consequently. In this model, the semantic proximity of justice and mercy emerges. Justice legitimizes the punishment of sinners, who must atone for their deed and possibly perform a compensatory act. Mercy, then, means the renunciation that results from the legal position and the forgiveness of sin so that the individual can remain in the community [52]. Although the context of this model is very narrow, it seems suitable for everyday life, since there are links for children especially in educational contexts (e.g. school). Against this background, we chose this model as a starting point for constructing a comprehension test based on picture short stories, which is explained in more detail in §2a(ii). Based on this model, in secular contexts, it is plausible that children would choose the semantic neighbour *just* (cf. table 1).

In model B, a situation begins with a person's distress, whereby it is insignificant who is to blame. Here, mercy is an attitude and act that seeks real solutions. Semantically, this model is closer to benevolence and kindness than to justice which means it has stronger links to the Bible and the Christian practice per se [52]. Owing to the general decrease in religious socialization of pupils [10], this model was secondary in the construction of our comprehension test (see above). Nevertheless, we assume that this model could be evoked in religious situations, which would be reflected in the choice of an appropriate semantic neighbour (cf. table 1).

In these considerations, aspects of context dependency can be identified: because the reference of the word is not an entity that can be singled out, the meaning of the word is changing when contextualizing it in various situations (cf. table 1). However, even though the meaning is context dependent, it can be differentiated or even contrasted with other actions or emotional impulses in these situations—a feature that is important for our setting. Derived from theological considerations, we can thus predict that depending on the situation, *mercy* will be differently conceived because it is the situational context (e.g. religious versus secular) that evokes associations with other words or actions and thus crucially steers the process of contextualization (*ad hoc* sense-making) of the religious word. We expect that the semantic neighbours ‘kind [lieb]’ and ‘just [gerecht]’ will be associated with ‘merciful [barmherzig]’ as their meaning corresponds more with a lifeworld familiarity. In our experimental setting (comprehension test), for the context variation, we will contrast religious with secular situations. We expect that both contexts will evoke different associations leading to another model for the meaning of *mercy* to be deployed with semantic neighbours that fit differently. In summary, the theological considerations provide clear models (table 1) allowing us to test the context dependency of religious abstract words, to our knowledge, for the first time.

The challenge of learning abstract (religious) concepts is that they cannot be pinned down to a few clearly distinctive semantic features that can be conveyed. As indicated above, a deep semantic structure of meaning can be derived from the theological models. It results in an action sequence that translates to a short plot. In order to grasp the concept of *merciful*, children probably need to be exposed to such a plot. For this purpose, joint reading of a book is particularly qualified wherein acting mercifully is a main subject to the protagonists' actions and motives clearly reflecting the semantic aspects (e.g. the Jonah story). By virtue of its dialogical form, joint book reading also allows opportunities to deepen and reflect on semantic aspects, to distinguish between similar concepts, to follow-up on ambiguities or to resolve misunderstandings with the other person in an interaction. Additionally, we regard linguistic strategies of emotionalization and perspectivation (cf. §§1a(i,ii) for further remarks) as supportive as they can anchor the abstract words with internal states. Following the literature specified in §§1a(i,ii), we expect that a crucial factor for a successful acquisition of *mercy* is that relevant semantic aspects of the word are not only mentioned, but linguistically enriched in such a way that they are perspectivized and emotionalized. In our view, this can only be achieved when both interactants actively contribute to the construction of meaning by the above-mentioned linguistic means (co-construction).

#### (i) Hypotheses

First, our hypotheses relate to children's conceptual understanding reflected in a picture selection test:

1a): owing to the contextualization, we assume that children more frequently recognize *mercy* as such in religious stories than in secular stories; and
1b): owing to its context dependency and tendency of convergence to non-religious adjacent concepts, we assume that children choose *kind* more often in religious contexts and the semantic neighbour *just* in secular contexts when not having opted for *merciful*.

Second, we propose a relation between the linguistic behaviour of the caregiver and the child (co-construction) in the reading situation and the child's conceptual understanding. Children who have experienced relevant semantic aspects emotionalized and perspectivized in a dialogic reading situation will gain a more enriched semantics of *mercy* demonstrated by using emotionalization and/or perspectivation and

2a): their use of enriched semantics will correspond to the behaviour of the caregivers also enriching their language––a process that we will refer to as co-construction;
2b): will use enriched semantics in their explanations during a subsequent picture selection test; and
2c): achieve higher scores in the receptive picture selection test than children who have experienced enrichment to a lesser extent in joint book reading.

## 2. Method

### (a) Study design and participants

In our pilot study, eight subjects (five females) aged 7–8 years participated with their caregivers. The study took place in a large city in the northwest of Germany. Six of the families were recruited via teachers of religious education at local primary schools. In accordance with university ethics procedures for research with children, parents provided written consent prior to their participation. Before visiting our laboratory (SprachSpielLabor), the families were sent a children's Bible [57] with the note to familiarize roughly with the Jonah story and that the concept of *mercy* was central in it.

The data collection consisted of three phases: (1) a dialogic reading situation between child and parent, which was realized as a non-participant observation; (2) a comprehension test to assess the child's understanding of *mercy* based on a set of picture cards; and (3) simultaneously, an interview on the caregiver's individual understanding of *mercy* and its family use. Everything was recorded with video cameras. In this paper, we focus on the reading situation and the picture test in detail, but also on the connections between both situations (1 and 2). Owing to the onset of the COVID-19 pandemic, we were forced to translate the data collection into an equivalent digital format. Therefore, two of the families conducted the reading situation and the picture test at home. Both were recorded via Zoom Video Communications, Inc. Care was taken to ensure the highest possible degree of comparability between the two settings.

#### (i) Dialogic reading situation

The reading situation took place in our laboratory room. The caregiver and child sat down on a sofa, whereby the former was not given any instructions on how to read the Jonah story with the child. Only the concept of *mercy* should be discussed.

#### (ii) Picture story test

The comprehension test took place directly after the reading situation. For warm-up, the experimenter began by asking the child what story she had just read with her parent and how she liked it. The experimenter then introduced the test by saying that a textbook for school was being written and that she needed the child's help by giving her opinion about the pictures. The test situation deviated from classical psycholinguistic comprehension tests as it was embedded in a pragmatic frame [58], which ascribed the role of an expert to the child and thereby encouraging her to narrate freely so that authentic and realistic verbal explanations could be uttered.

The test itself was based on five sets of picture cards created especially for the pilot study. The first set of picture cards aimed to familiarize the child with the test format without depicting *mercy* on it. The beginning of each picture story was told accompanied by an opening image whereupon four alternative endings were told and presented on pictures. Finally, the child was asked to choose the most appropriate picture and to briefly explain her choice and also her reasons for the non-chosen pictures. After familiarization, two religious stories (Citizens of the City of Nineveh and The Tax Collector Zacchaeus) and two secular stories with an everyday character (within the contexts of home and school) were presented to the child. The alternative endings followed our religious model A (cf. §1b) and a psycholinguistic conceptualization into semantically close and distant concepts from *mercy* and represented the following behaviours of the protagonists in the stories: *merciful* (target item), *just* and *kind* (both semantically close distractors), and *hard-hearted* (semantically distant distractor).

Each opening image depicted a person who is burdened with guilt and consequently, *mercy* is always an act of forgiveness. For example, in one of our religious picture stories, the initial situation is as follows: *In the city called Nineveh total chaos reigns. The inhabitants are fighting with each other, hurting other people and setting houses on fire. God is watching everything from heaven.* Therefore, a merciful action was defined according to the semantic aspects (cf. model A in table 1) that were integrated at the target picture and the experimenter's verbal description.

To prevent the children's choices from always selecting similar pictures, the test question targeted *merciful* and *just* each twice, which functioned as mutually close distractors. In this paper, only the results according to the target item *merciful* are reported, which means that the analysis is based on a maximum of two sets of picture cards. The presentation of the stories, the order of the alternative endings, and the test question for *merciful* or *just* were completely randomized.

### (b) Data coding

The video data were transcribed and coded using the linguistic annotation software ELAN [59].

#### (i) Receptive abilities in the picture selection test

To assess the children's receptive abilities of *merciful*, we analysed the children's nonverbal answers, i.e. which picture the children chose in response to the test question. We refrained from a binary coding in right or wrong and used the following multi-level ranking system: selection of the antonym *hard-hearted* (0); clear selection of the semantically close distractor *just* / *kind* (1); ambiguous selection, i.e. hesitation between the target item and a semantic neighbour or self-correction (2); clear selection of the target item *merciful* (3). By means of this coding, we were able to assess vagueness and overlaps of choices. The value reported in the results (§3a,d) is an average value of two test questions.

#### (ii) Production of semantic aspects and its enrichment by emotionalization and perspectivation

In the reading situation, coding was done from the time the story was introduced until caregiver and child decided that the dialogic reading was over. In the picture test, coding started after the test question for *merciful* and included the child's explanations on why she had not chosen another picture card.

In the first step of our analysis, we focused on the semantic aspects produced by the children (explanations in the picture test; dialogic reading) and their caregivers (dialogic reading). Following two specific models (table 1), we assigned the childrens' and caregivers' utterances to the corresponding semantic aspects. For example, in the picture test, a child said about the inhabitants of the city of Nineveh ‘Because they haven't done anything good.’, which corresponds to the first semantic aspect of a person's guilt (cf. table 1). In the picture test, the analysis was carried out independently of the correct receptive selection of the target item *mercy*, i.e. even if the child had chosen the close distractor *just*, the experimenter asked for the reasons of her choice (‘Why do you think the mother in this picture is merciful?’). In a second step, we examined whether and how often these semantic aspects were enriched by means of emotionalization and perspectivation.

Linguistic means that contributed to an emotionalization of an utterance became evident through prosody (e.g. a sad voice), emotionally valent words (e.g. *destroy* as negatively valenced) and emotional states that had to be inferred on the basis of an action sequence. Also superlatives or exaggerations as well as emotional exclamations (interjections) expressed emotionality in language (cf. [60]).

Linguistic means that contributed to perspectivation were the use of direct and indirect speech, prosodic means such as voice modulation (cf. [42,43]) and the use of mental state words (e.g. volitional verbs) that highlights a speaker's perspective [43]. Also questions that encouraged the child to think about certain intentions of a protagonist, subjective judgements, self-referential comments or generalizations (cf. [41]) fell into this category.

The coding was based on the number of total occurrences in the reading situation or in the picture test.

## 3. Results

### (a) Contextual conceptual understanding

All analyses were computed in R [61].

First, we report children's conceptual understanding in the picture selection test and how the performance differs with respect to the different contexts.

In the picture test, we looked at which pictures the children chose across contexts: on average, their decisions ranged between the target object and a semantic neighbour (median (Mdn) = 2, range = 1–2, interquartile range (IQR) = 0.61). In the religious context, in 57.14% of the cases, the item selection of the target *mercy* was completely correct, whereas in the secular contexts, it did not occur a single time. As hypothesized in hypothesis 1a, a comparison based on the scores obtained in the comprehension test (rank scores cf. §2b(i)) showed that the children scored significantly higher in the stories about religious contexts (Mdn = 3, range = 1–3, IQR = 1.5) than in the secular stories (Mdn = 1, range = 1–2, IQR = 0), i.e. they selected a semantic neighbour (rank score = 1) less often and the target item (rank score = 3) more often in these picture stories (*W* = 15, *p* = 0.02, *r* = –0.60).

Next, we analysed the semantic proximity expressed by the choice of distractors, assuming that the children synonymized *merciful* with *kind* or *just* depending on its context (hypothesis 1b). Considering only the distractors across all contexts, 70.0% of the children's choices fell on the picture that depicted *kind* and 30.0% on the picture that visualized *just*. Overall, the children selected the distractor *kind* (Mdn = 1, range = 0–2, IQR = 0.25) as frequently as *just* (Mdn = 0, range = 0–1, IQR = 1), *W* = 21, *p* = 0.24, *r* = –0.29. As expected, the children selected the semantic neighbour *just* more often in the secular context than in the religious context, ($\chi_{1}^{2}=3.81$, *p* = 0.05) which is in accordance with our model A of *mercy* in which the semantic aspects of forgiveness are central (see §1b for further remarks). Contrary to our hypothesis, an equal number of children opted for the adjacent concept *kind* in both contexts, ($\chi_{1}^{2}=0.29$, *p* = 0.59). That is, regardless of the (religious) context, the children conceived the semantic aspects of loving care of mercy (cf. model B).

### (b) Co-constructing meaning by emotionalization and perspectivation during reading

For the following analysis, we assumed that caregiver and child would co-construct the word's meaning in duet with each other during dialogic reading of a biblical story (hypothesis 2a). More specifically, we predicted a correlation between children's and caregiver's semantic enrichment.

For the analysis of the co-construction and owing to the small sample, we chose the rank-based correlation coefficient Kendall's tau (*τ*) to account for the non-normally distributed data and shared ranks. We found a significant positive correlation between caregiver's and children's use of emotionalization (*τ* = 0.53, *p* = 0.05) meaning that the more a caregiver enriched semantic aspects of *mercy* by means of emotion, the more the child did so as well or vice versa. It is important to emphasize that we did not look for an overlap (e.g. caregiver and child both using emotionally valent words) but calculated the correlations as a general enrichment of emotionalization (e.g. caregiver uses a word that is emotional valenced whereas the child uses exaggerations).

With regard to perspectivation, no relation between the caregiver's and children's behaviour was found (*τ* = 0.23, *p* = 0.25). We found that only two of eight children made use of semantic enrichment by the means of perspectivation in comparison to five adults.

In a further step, we analysed whether the simultaneous enrichment by both perspectivation and emotionalization was coordinated between caregiver and child and found no correlation (*τ* = 0.23, *p* = 0.25).

Interestingly, in our sample, the five caregivers who used perspectivation also applied emotionalization during dialogic reading whereas there were three parents who did not enrich the semantic aspects at all.

### (c) Production of enriched semantics in the picture test in relation to co-construction

For the following analysis of childrens' explanations for their decisions in the picture selection test, we focus on whether they used semantic aspects and how often they were enriched by means of emotionalization and perspectivation.

Seven out of eight children used emotionalization or perspectivation (Mdn = 3.5, range = 0–7, IQR = 3.25) during the picture test. We further analysed whether children's use of emotionalization and perspectivation was related to what they experienced during dialogic reading, i.e. the way their caregivers used the semantic enrichment. We found a positive but only marginally significant correlation when perspectivation and emotionalization was used simultaneously (*τ* = 0.46, *p* = 0.06). This finding verifies our hypothesis 2b.

### (d) Relations between the co-construction and the conceptual understanding in the picture test

In a final step, we analysed to what extent the co-constructed and enriched semantics were related to children's scores achieved in the receptive picture test overall, but also separated for religious and secular stories. In the picture test, children's decisions ranged on average between the target object and a semantic neighbour (Mdn = 2, range = 1–2, IQR = 0.6). As a measure of co-construction, we used measurements reported in §2c.

For the overall test score, we neither found a correlation with the use of perspectivation (*τ* = 0.05, *p* = 0.44), nor emotionalization (*τ* = 0.15, *p* = 0.33), nor perspectivation and emotionalization that occurred simultaneously (*τ* = 0.05, *p* = 0.44). This analysis was also not significant when considering the religious and secular contexts separately. Overall, this falsified our hypothesis 2c.

## 4. Discussion

In our pilot study, for the first time to our knowledge, we account for the acquisition of religious abstract words in relation to the input children receive. For this, we studied a small sample of primary school pupils. We chose the word mercy as an object of investigation because it is a prototypical religious word which anchors emotionalization and perspectivation through language. Our starting point was that the mere naming of semantically relevant aspects is not sufficient to experience the profound meaning of it. Instead, an enrichment by the related linguistic means is necessary but has to be constructed by child and caregiver together. This claim links to recent approaches suggesting that in addition to intrapersonal meaning construction, the semantics of an abstract word needs to be established interpersonally [3,62]. So far, no study in language acquisition considered the interpersonal meaning construction, let alone its relation to the intrapersonal semantics development. In this respect, our study contributes a systematic mix of experimental settings and codings to account for this relation.

In our investigation, first, we focused on the contextualization and discovered that as hypothesized, in the religious context, children's performance was best. Surprisingly, the children were not able to generalize their concept to secular contexts and chose exclusively semantically close distractors instead. This provides support for *merciful* being strongly religiously bound. Our finding extends current research to important insights into contexts dependencies of abstract words. Clearly, contexts of use have to be a methodological aspect when studying children's semantic development. Because *mercy* is so deeply religiously tied, we have to critically ask whether embedded in a religious context, our dialogic reading situation with the biblical story should have been supplemented by an additional secular context to elicit better generalization effects in the picture test. Using the word in a variety of contexts and situations in duet with their caregiver (and other conversational partners) will foster a deep knowledge of the concept. This mechanism is valid for the acquisition of all abstract concepts, but is particularly relevant for religious concepts that are not used in everyday contexts nowadays, so that a general basic link is missing which needs to be established first. In future study designs, this link for acquisition could be provided by contrasting mercy with semantically close and non-religious words like *just* or *kind* even during dialogic reading. At the same time, conceptual similarities, differences and overlaps could emerge more transparently for children, and consequently, could be addressed in the comprehension test.

To account for children's semantic knowledge about the word *mercy*, we applied theological models––an innovative method resulting from an interdisciplinary collaboration. We had designed our picture card test according to a specific model of forgiveness (cf. table 1, model A), in which the semantically close and more appropriate neighbour was *just*. In the secular context, the majority of the children opted for *just*, which was in line with our hypothesis owing to the underlying context dependency of *mercy*. Alternatively, *kind* was selected equally frequently by the children in both contexts, which contradicted our hypothesis in which we assumed a primacy of the religious context. However, interestingly, not a single child chose *just* in the religious context. From these results we conclude that for children, our model might not clearly distinguish forgiveness from loving care. So it rather suggests a broader conceptual association that involves both models, especially when secular contexts are involved. Another interpretation is a context-dependent understanding comprising *merciful* and *kind* together when religious content is prominent. Clearly, further research is necessary to investigate whether model A of forgiveness can be applied to children at all as it seems that it creates an artificial conceptual narrowing.

In a second step of our investigation, we focused on the joint reading. Here, assuming that the mention of the relevant aspects of *mercy* is not sufficient but needs to be enriched, we analysed the verbal behaviour of both, the child and the caregiver for the semantic enrichment. We found a positive relation between the partners' behaviours for emotionalizing. With our measure, we captured the degree of emotionalization tied to the semantic aspects across the entire reading situation and did not focus on emotional alignment in consecutive turns at a local level or even on the type of emotional consonance, e.g. positive or sad [63]. Even if our study design (owing to the lack of a comparative situation, e.g. without abstract words) does not allow ruling out that a certain degree of emotional alignment was at work here, the above-mentioned causes may indicate that we have only measured emotionalization.

Although perspectivation is a central aspect in the meaning of *mercy*, in our sample, it was not applied as prominently as the emotional component. This could be explained by the word form containing *heart* and, thus, expressing a strong emotional valence [17]. At the same time, the semantic aspect that a person is figuratively touched at the heart by another's distress draws from taking another's perspective [44].

In our data, it appears that the caregiver more often gave a linguistic impulse that induced perspectivation than emotionalization: in the first place, this allowed children to perceive another person's distress and second, to recognize underlying motives and feelings. In contrast with caregivers, children very rarely used means of perspectivation, but rather emotionalized, demonstrating that they empathized with another's distress. Overall, our data yielded very individual solutions of the investigated caregiver–child dyades for the use of semantic aspects and linguistic enrichment. That is, dialogic reading *per se* was realized in a variety of ways: some caregivers read the story completely and engaged then in a dialogue with their child. Others interrupted the reading flow at some points for a short conversation; yet others almost exclusively read aloud. We thus propose that the parents perceived their role in the situation differently: some may have perceived themselves more as an expert, whereas a few only took on the role of a reader and added little to the Bible content. What becomes obvious is that the Bible can be read differently than other (picture) books. The authority that this book communicates led some caregivers to rely on it, without their own contribution in form of elaboration or interpretation. We consider this point as an important critical point to our method. Accordingly, we have to limit our conclusions: It is not only the caregiver's familiarity with the concept of mercy that shaped the observed interaction, but also their role in the reading routine as well as the reading routines in the family that go beyond the book content [64].

In a further step, we identified relation between the co-construction in reading and the children's verbal behaviours that led to enriched semantics found in the test with a positive trend for all means of enrichment. Interestingly, we revealed that semantic aspects co-constructed in the reading situation are directly related to those addressed in the test, especially when the semantic aspects were enriched by emotionalization and perspectivation simultaneously. For expressing initial understanding of *mercy*––a word that is emotionally valenced and at the same time requires and involves perspectivation––children used both linguistic strategies that they also experienced during joint reading. Children who did not co-construct during reading also did not use enrichments in the test situation. The question of how this enrichment contributes to the acquisition of the word remains unanswered. In fact, we could not find any correlation between the co-construction in reading and the test scores. We can only argue that the picture test can be considered as a further learning situation in which the child deepens their ability to distinguish the semantic concept of *mercy* from closely related concepts to mark relevant aspects. For such learning, the use of enrichment appears to be meaningful.

Overall, our pilot study reveals that emotionalization and perspectivation––leading to successful semantic enrichment of abstract words [20,28,38–40]––are also applied in both children and caregivers when using religious abstract words. Based on theological consideration and applying models accounting for the word's semantics, we were able to show that children apply the concept of *mercy* depending on the context. The context dependency is an interesting aspect that needs to be followed up in future research, also for non-religious abstract words.

## Data Availability

There is no data associated with this article.
